# Genetic modelling of the weekly weight of Japanese quails using orthogonal Legendre polynomials

**DOI:** 10.5194/aab-69-441-2026

**Published:** 2026-08-20

**Authors:** Arzu Üçtepe, Antonios Kominakis

**Affiliations:** 1 Department of Animal Science, Isparta University of Applied Sciences, Isparta, 32200, Turkey; 2 Department of Animal Science, Agricultural University of Athens, Iera Odos 75, 11855 Athens, Greece

## Abstract

In this study, Legendre polynomial random regression models (RRMs) of orders 2 to 6 were applied to estimate genetic parameters (heritability coefficients and genetic correlations) for weekly body weight (5745 records) from hatch up to 9 weeks of age in 575 Japanese quails (423 females and 152 males). Based on the Bayesian information criterion (BIC), the optimal RRM was of the fifth order and incorporated nine heterogeneous residual variance classes. The model demonstrated high predictive accuracy, yielding a symmetric mean absolute percentage error (SMAPE) of 2.6 % and a CV(RMSE) of 5.0 %. Heritability estimates ranged from 0.25 (at hatch) to 0.15 (at day 63), peaking at day 21 ( $h^{2}\approx 0.30$ ). Genetic parameter estimates from the optimal RRM were highly consistent with those from a multivariate analysis, showing that genetic correlations were highest between adjacent ages and decayed progressively as the time interval increased. Eigenfunction decomposition of the additive genetic covariance matrix identified two principal modes of variation: the first eigenfunction (84.8 %) represented uniform shifts across the entire growth trajectory, whereas the second (10.2 %) captured a genetic trade-off between early and late growth phases. Simulated selection targeting the top 10 % of birds based on estimated breeding values (EBVs) at early (1–14 d), middle (21–35 d), and late (42–63 d) growth stages differentially altered the genetic response curves, demonstrating the capacity to tailor selection strategies to specific breeding objectives. Overall, selection between 21 and 35 d of age provided the most balanced genetic response across the growth trajectory, identifying this period as the optimal selection window for Japanese quail breeding programmes.

## 1 Introduction

In avian species such as Japanese quail (*Coturnix japonica*), body weight is a primary indicator of growth potential, feed efficiency, and slaughter value (Sokhan et al., 2025). The species has gained increasing attention in both experimental and commercial contexts due to its short generation interval, high reproductive rate, and utility as a model organism in developmental biology and genetics (Minvielle, 2004). Historically, genetic analysis of growth has relied on univariate or multivariate models fitted to body weights at discrete time points. While informative, such approaches treat measurements at different ages as separate traits, ignoring the underlying biological continuity and potential autocorrelation between time-adjacent weights and overlooking the heterogeneity of genetic and residual (co)variances across age (Meyer, 1998). To address these limitations, random regression models (RRMs) have become increasingly popular in animal breeding, particularly for modelling longitudinal or repeated records obtained in various time intervals within a given timescale, including time intervals that were not sampled (Berry et al., 2003; Kominakis et al., 2001; Kranis et al., 2007; Schaeffer and Dekkers, 1994; Schaeffer, 2004; Van der Werf et al., 1998).

RRMs allow each animal's phenotype to be modelled as a continuous function of time, where individual differences are captured through random regression coefficients. The approach enables the estimation of genetic (co)variance functions across all time points, providing a more biologically coherent view of trait development (Kirkpatrick et al., 1994; Meyer and Hill, 1997). RRMs are also flexible in that they (i) do not require prior assumptions about the shape of the growth curve and (ii) can accommodate reduced-order fits, yielding smoothed covariance structures with fewer parameters when appropriate.

A crucial component of RRMs is the selection of an appropriate set of basis functions to model time. Among various choices, orthogonal Legendre polynomials (LPs) have proven particularly effective due to their mathematical properties. First introduced for modelling growth trajectories by Kirkpatrick et al. (1990) and later popularized in animal breeding by Meyer (1998), LPs offer orthogonality, which minimizes collinearity among regression coefficients, leading to stable estimation and interpretable covariance structures, with relatively few parameters (Nobre et al., 2003). This methodological advantage has made LP-based RRMs a preferred approach in many genetic evaluations of longitudinal traits in animal breeding (Jamrozik et al., 1997; Schaeffer and Dekkers, 1994; Van der Werf et al., 1998).

Among avian species, RRMs have been effectively implemented in chickens, including layers and native breeds (Guo et al., 2020; Miyumo et al., 2018; Tongsiri et al., 2020), turkeys (Rafat et al., 2011), and ostriches (Nel et al., 2025). Several studies have applied RRMs to analyse growth in quail populations (Karami et al., 2017; Mota et al., 2018; Taroco et al., 2019). Although these studies demonstrated the utility of RRMs for modelling longitudinal growth data, the full potential of this methodology for characterizing individual growth trajectories, estimating age-specific genetic parameters, and identifying biologically meaningful windows for stage-specific selection remains unexplored. Given that growth dynamics and genetic architecture are population-specific, further investigations are needed to optimize the application and interpretation of RRMs across diverse quail populations.

The present study aims to provide a unified analytical framework that extends beyond conventional RRM analyses by integrating covariance eigenfunction decomposition and stage-specific estimated breeding value (EBV)-based selection scenarios. Eigenfunction decomposition was used to biologically interpret the temporal structure of additive genetic variation, whereas age-specific selection scenarios were employed to evaluate how the timing of selection influences longitudinal genetic response. By combining these approaches, the study provides a more comprehensive understanding of the genetic architecture underlying growth trajectories and offers biologically meaningful and practically relevant insights into age-dependent selection strategies in the Japanese quail.

## 2 Material and methods

### 2.1 Ethical approval

The present study was conducted using phenotypic records of weekly body weights obtained from Japanese quails reared at the Quail Breeding Unit, Isparta University of Applied Sciences. Therefore, it did not require any ethical approval.

### 2.2 Material

#### 2.2.1 Body weight records

A total number of 5745 weekly body weights of 575 (423 females and 152 males) animals were made available from hatch (day 0) to the 63rd day of age. By 63 d of age, birds had largely attained mature body weight, and growth had substantially plateaued, allowing the complete growth trajectory from hatch to near maturity to be modelled. A total of 570 animals had records at all age classes ( n=10 ), while 5 animals had records at 9 age classes.

#### 2.2.2 Pedigree

The pedigree comprised 705 animals, of which 575 (81.6 %) had BW (body weight) records. The pedigree included 365 parents with recorded offspring, consisting of 123 sires and 242 dams, providing substantial genetic connectedness among individuals. All animals with records had at least one known parent. Pedigree information extended to the grandparental generation, with 348 animals having a known paternal grandsire, 358 a known maternal grandsire, 335 a known paternal grand dam, and 335 a known maternal grand dam. Furthermore, 84.4 % of animals in the pedigree were connected through their own records or through parental and sibling relationships. Overall, the pedigree structure indicated a satisfactory level of connectedness and depth for the estimation of genetic parameters. Pedigree structure statistics were obtained from the pedigree summary generated by WOMBAT (Meyer, 2007).

#### 2.2.3 Statistical analyses

The first set of analyses fitted RRMs for individual animal effects on orthogonal polynomials of age at measurement. Polynomials chosen were the LPs defined for the range of - 1 to + 1 (Kirkpatrick et al., 1990). LPs ranging from second to sixth order were considered in the present study, using the following model:

1 $$\begin{array}{rl} y_{ij} & =F_{ij}+\sum_{k=0}^{p}\varphi_{k}\left(t_{j}\right)\beta_{k}+\sum_{k=0}^{p}\varphi_{k}\left(t_{j}\right)a_{ik}\\ & +\sum_{k=0}^{p}\varphi_{k}\left(t_{j}\right)p_{ik}+e_{ij}, \end{array}$$

where $y_{ij}$ is the observed value of the trait for individual i at day of measurement $t_{j}$ ; p is order of the LP; $F_{ij}$ is the fixed effects pertaining to $y_{ij}$ with egg weight (average = 12.2 g, SD = 0.9 g) and cage (129 classes) as covariates and sex (two classes: females and males), generation (three classes: 1–3), and cage incubation time (four classes: days 18–21 of incubation) as class effects; $\varphi_{k}\left(t_{j}\right)$ is the k th LP evaluated at standardized time $t_{j}\in [-1,1]$ ; $\beta_{k}$ is the fixed regression coefficient for polynomial k (population mean trajectory) of the fifth order; $a_{ik}$ is the random regression coefficient for the additive genetic effect of animal i for polynomial k ; $p_{ik}$ is the random regression coefficient for the permanent environmental effect of animal i for polynomial k ; and $e_{ij}$ is residual error.

The $t_{j}$ day at measurement was rescaled to the range of the orthogonal functions [-1,1] according to the formula given by Kirkpatrick et al. (1990). For the vector of days 0 to 63, the vector of standardized ages onto the interval [-1,1] was $a^{*}$ = [ - 1.000, - 0.778, - 0.556, - 0.333, - 0.111, 0.111, 0.333, 0.556, 0.778, 1.000].

Additive genetic and permanent environmental regressions were modelled with the same polynomial order ( p ) throughout to ensure comparable flexibility in describing age-dependent variation and to avoid confounding differences between variance components with differences in model parameterization. The residual error variance was allowed to be heterogeneous, changing with the day of measurement to better capture age-specific variability. To this end, nine residual error classes were specified: class one with 1–7 d, class two with 8–14 d, class three with 15–21 d, class four with 22–28 d, class five with 29–35 d, class six with 36–42 d, class seven with 43–49 d, class eight with 50–56 d, and class nine with 57–63 d. The optimal RRM identified using the joint dataset (both sexes combined) was also applied separately to model sex-specific genetic trajectories over age. A two-sided Wilcoxon signed-rank test for paired samples (10 paired observations at the same ages) was then applied to assess whether heritability ( $h^{2}$ ) and permanent environmental effects ( $c^{2}$ ) differed between sexes. Genetic analysis was complemented by performing a multivariate (MV) – a 10-variate – analysis in which each age class was treated as discrete trait(s). This analysis allowed for comparison of estimates of genetic parameters with the estimates obtained from the optimal RR model. Fixed and random effects were defined as in the RR models. All pedigree information available was included in all analyses in order to minimize bias due to selection and to increase the accuracy of estimation.

LP-based RRMs and MV analyses were performed using WOMBAT software (Meyer, 2007). For the RRMs, convergence was achieved using the Average Information REML (AI-REML) algorithm (aireml option). For the MV analyses, several alternative optimization strategies were evaluated. Convergence was ultimately achieved by modifying the average information matrix through Cholesky decomposition using the -modaim3 option.

#### 2.2.4 Model selection criteria

Model fit was assessed using the Akaike information criterion (AIC; Akaike, 1974) and the Bayesian information criterion (BIC; Schwarz, 1978), calculated as

2AIC=-2log⁡L+2k3BIC=-2log⁡L+kln(N),

where log L is the maximum likelihood of the model, ( k ) is the number of estimated parameters, and ( N ) is the number of observations. Note that AIC favours more complex models, while BIC promotes parsimony due to its stricter penalty for model complexity (Chakrabarti and Ghosh, 2011).

#### 2.2.5 Model prediction accuracy

We used two metrics to assess the model prediction accuracy: the deviation between the predicted and observed values. For this task, the symmetric mean absolute percentage error (SMAPE) and the coefficient of variation in the root mean square error (CV(RMSE)) were employed, calculated as follows (Chicco et al., 2021):

4SMAPE=1n∑i=1nwP-wOwP+w/2×100,5CV(RMSE)=RMSEwO‾×100,

with

6 $$\mathrm{RMSE}=\sqrt{\frac{1}{n}\sum_{i=1}^{n}(w_{P}-w_{O}{)}^{2}},$$

where $w_{O}$ is the observed weight, $w_{P}$ is the predicted weight, and N is the number of fitted points.

#### 2.2.6 Estimation of genetic eigenfunctions

We performed eigen decomposition on the additive genetic covariance matrix $G\in R^{\left(p+1\right)x(p+1)}$ to characterize the principal modes of genetic variation underlying the growth trajectory.

7 $$G=V\Lambda V^{\prime },$$

where $V=[v_{1},v_{2},\ldots ,v_{p+1}]$ is the matrix of eigenvectors, and $\Lambda =\mathrm{diag}(\lambda_{1},\lambda_{2},\ldots ,\lambda_{p+1})$ are eigenvalues.

Then, the r th eigenfunction $\psi_{r}\left(t\right)$ is

8 $$\psi_{r}\left(t\right)=\varphi {\left(t\right)}^{\prime }v_{r}=\sum_{k=0}^{p}\varphi_{k}(t_{j})v_{kr},$$

where $\varphi_{k}(t_{j})$ is the normalized Legendre polynomial of degree k evaluated at age $t_{j}$ rescaled to [-1,1] , and $v_{kr}$ represents the components of the eigenvectors of G .

#### 2.2.7 Estimation of breeding values (EBVs)

EBVs at a particular age $t_{j}$ for animal i were computed from its random regression coefficients $a_{i}={\left[a_{i0},a_{i1},\ldots a_{ip},\right]}^{\prime }$ as

9 $${\mathrm{EBV}}_{i}(t_{j})=\sum_{k=0}^{p}\varphi_{k}(t_{j})a_{ik},$$

where $a_{ik}$ is the k th Legendre coefficient for animal i , and $\varphi_{k}(t_{j})$ is the normalized Legendre polynomial of degree k evaluated at age $t_{j}$ rescaled to [-1,1] .

#### 2.2.8 Evaluation of selection at different growth stages based on EBVs

A hypothetical selection scenario was evaluated by selecting the top 10 % of birds based on EBVs within three growth phases: early (1–14 d), intermediate (21–35 d), and late (42–63 d). The top 10 % selection pool ( n=68 ) was derived from a number of 681 pedigree animals (out of 705 total) with available EBVs based on their genetic connectedness, including both phenotyped birds ( n=575 ) and unrecorded relatives. The overlap among selected groups was then used to assess the consistency of selection across growth stages and to identify animals with persistent or phase-specific genetic merit.

## 3 Results

### 3.1 Model selection and model fit

As shown in Table 1, increasing the LP order from 2 to 6 progressively improved model fit, yielding higher log L values and lower AIC values, while BIC decreased up to the fifth order. The largest gains occurred at lower orders, with improvements tapering off at higher degrees. While the sixth-order model maximized log L , its marginal improvement over the fifth-order model did not justify the estimation of 14 additional parameters (65 vs. 51). Furthermore, the fifth-order model minimized the BIC, indicating an optimal compromise. Consequently, the fifth-order model was selected for all subsequent analyses as it provided the best balance between goodness of fit and parsimony.

**Table 1:** Values of maximum likelihood (log L ) and model selection criteria (AIC and BIC) for varying (from 2 to 6) degree of the LPs used to model the quail data.

Order of	Number of parameters	Maximum	1/2 AIC	1/2 BIC
LP ( p )	estimated ( k )	Log L		
2	21	- 16305.8	16326.8	16396.6
3	29	- 15827.2	15856.2	15952.6
4	39	- 15587.9	15626.9	15756.7
5	51	- 15493.4	15544.4	15714.1
6	65	- 15448.8	15513.8	15730.0

**Figure 1:**
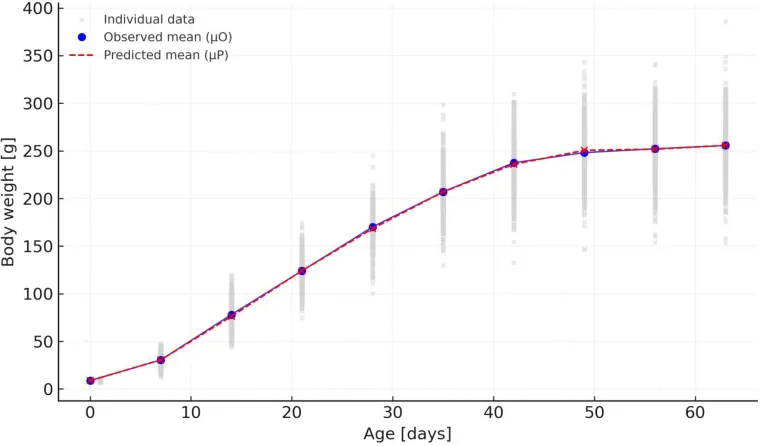
Observed (blue circles) and predicted mean body weight (x red) across ages. The dashed red line illustrates the fifth-order LP curve. Grey dots show individual measurements.

The fifth-order LP accurately described quail growth, as shown in Fig. 1 and Table 2. At all ages, there were no significant differences between observed ( $\mu_{O}$ ) and predicted ( $\mu_{P}$ ) mean weights, as their 95 % confidence intervals overlapped (Table 2). The model's predictive performance was also confirmed by low SMAPE (2.62 %) and CV(RMSE) (4.98 %) values.

**Table 2:** Number of observations ( N ) as well as observed ( $\mu_{O}$ ) and predicted weight means ( $\mu_{P}$ ) with 95 % CI limits in 10 age classes (d) for Japanese quails.

		Weight (g)	
Age (days)	N	$\mu_{O}$ (95 % CI limits)	$\mu_{P}$ (95 % CI limits)
0	575	8.7 (8.7–8.8)	8.7 (8.7–8.8)
7	575	30.5 (30.0–31.0)	30.5 (30.0–31.0)
14	570	78.0 (76.7–79.2)	76.2 (75.2–77.3)
21	575	124.0 (122.7–125.3)	124.1 (122.7–125.4)
28	575	170.1 (168.6–171.6)	168.4 (167.0–169.8)
35	575	207.0 (205.1–208.9)	206.9 (205.5–208.4)
42	575	237.5 (235.2–239.8)	235.9 (234.2–237.6)
49	575	248.1 (245.8–250.4)	250.5 (248.5–252.6)
56	575	252.2 (249.9–254.6)	251.8 (249.5–254.0)
63	575	255.6 (252.6–258.7)	255.7 (252.8–258.7)

Predicted weights ( $\mu_{P}$ ) were derived from the optimal fifth-order RRM.

### 3.2 Age-specific heritability estimates for both sexes

Heritability estimates obtained from the optimal RRM and MV analyses were generally consistent, both showing a peak around 21 d of age followed by a decline at later ages (Table 3). However, the MV analysis tended to yield slightly higher heritability estimates after 42 d. Similarly, permanent environmental effects ( $c^{2}$ ) followed comparable trends in both approaches, although RRM estimates were generally higher at later ages. Overall, the close agreement between methods supports the robustness of the results.

**Table 3:** Estimates of heritability and ratio of permanent environmental effects to total phenotypic variance ( $c^{2}$ ) from the optimal RRM and MV analysis (standard errors of estimates in parentheses).

	RRM		MV	
Age (days)	$h^{2}$ (SE)	$c^{2}$ (SE)	$h^{2}$ (SE)	$c^{2}$ (SE)
0	0.25 (0.07)	0.75 (0.08)	0.27 (0.08)	0.37 (0.07)
7	0.16 (0.06)	0.84 (0.06)	0.18 (0.07)	0.69 (0.07)
14	0.18 (0.05)	0.56 (0.05)	0.24 (0.08)	0.45 (0.07)
21	0.30 (0.07)	0.70 (0.07)	0.30 (0.07)	0.55 (0.07)
28	0.22 (0.06)	0.45 (0.05)	0.26 (0.08)	0.42 (0.07)
35	0.19 (0.06)	0.46 (0.06)	0.16 (0.07)	0.38 (0.07)
42	0.14 (0.06)	0.58 (0.06)	0.17 (0.07)	0.51 (0.07)
49	0.11 (0.06)	0.69 (0.06)	0.20 (0.07)	0.56 (0.07)
56	0.14 (0.06)	0.80 (0.07)	0.23 (0.08)	0.66 (0.07)
63	0.15 (0.07)	0.78 (0.07)	0.22 (0.08)	0.62 (0.08)

### 3.3 Genetic and phenotypic correlations across ages for both sexes

**Figure 2:**
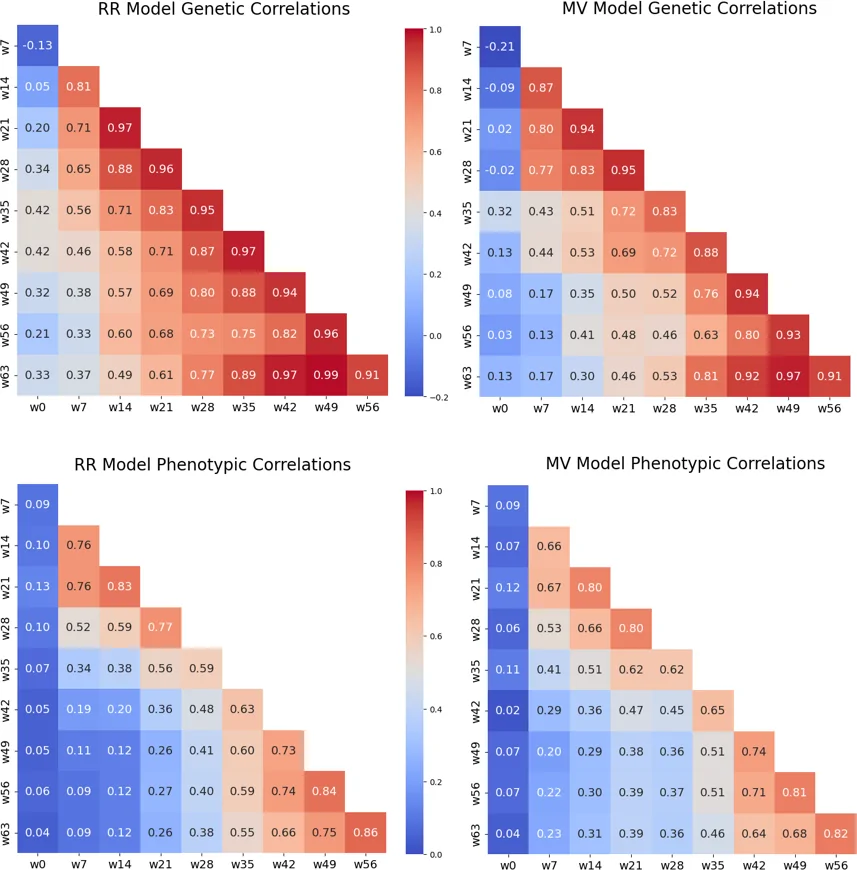
Estimates of genetic correlations (upper part) and phenotypic correlation (lower part) by age (left: RR; right: MV; (standard errors of genetic correlations are in the range of 0.19–0.29, and standard errors of phenotypic correlations are in the range of 0.03–0.07).

Figure 2 illustrates the genetic and phenotypic correlations for the trait across the time trajectory, estimated using RRM and MV analyses. In both models, genetic correlations showed a distinct pattern of increasing strength with age with weak or even negative correlations between early time points and markedly stronger between later time points. A clear temporal pattern emerged with correlations being highest between adjacent ages and progressively decreasing as the interval between measurements increases. Phenotypic correlations closely followed the same temporal trend as genetic correlations, with higher correlations at later ages and between adjacent time points. Comparing the models, the RRM consistently yielded higher correlations than the MV analysis for both genetic and phenotypic estimates and provided smoother estimates over time.

### 3.4 Sex-by-age specific estimates

Table 4 presents estimates of heritability ( $h^{2}$ ) and permanent environmental effects ( $c^{2}$ ) from the optimal RRM in males and females. Based on the Wilcoxon signed-rank test, heritability estimates ( $h^{2}$ ) did not differ significantly between sexes ( p=0.4922 ). In contrast, permanent environmental effects ( $c^{2}$ ) were found to significantly differ ( p=0.0371 ) between sexes. Nevertheless, given the small sample size (particularly within the male group) and the associated elevated sampling variance of estimates, these findings must be treated with caution.

**Table 4:** Estimates of heritability ( $h^{2}$ ) and permanent environmental effects ( $c^{2}$ ) from the optimal RR model in male and female Japanese quails (standard errors of estimates in parentheses).

	Males			Females		
Age	N	$h^{2}$	$c^{2}$	N	$h^{2}$	$c^{2}$
0	152	0.195 (0.211)	0.801 (0.222)	423	0.341 (0.097)	0.655 (0.107)
7	152	0.213 (0.173)	0.787 (0.176)	423	0.272 (0.098)	0.728 (0.101)
14	152	0.114 (0.142)	0.643 (0.139)	418	0.278 (0.079)	0.457 (0.074)
21	152	0.189 (0.179)	0.769 (0.180)	423	0.419 (0.104)	0.572 (0.104)
28	152	0.238 (0.129)	0.444 (0.124)	423	0.323 (0.083)	0.370 (0.076)
35	152	0.379 (0.148)	0.359 (0.140)	423	0.303 (0.084)	0.292 (0.075)
42	152	0.447 (0.162)	0.363 (0.155)	423	0.336 (0.093)	0.335 (0.085)
49	152	0.412 (0.140)	0.325 (0.131)	423	0.383 (0.097)	0.391 (0.090)
56	152	0.517 (0.171)	0.446 (0.170)	423	0.464 (0.106)	0.436 (0.105)
63	152	0.457 (0.185)	0.542 (0.240)	423	0.359 (0.105)	0.561 (0.139)

**Figure 3:**
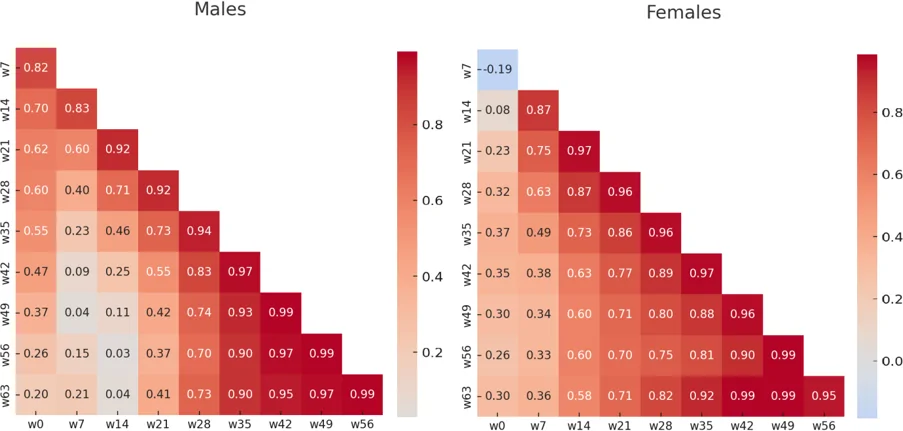
Heatmaps of genetic correlations between weekly body weights estimated from the optimal RRM in male (left) and female (right) Japanese quails (standard errors of genetic correlations are in the range of 0.28–0.68 and 0.15–0.25 for males and females, respectively).

Figure 3 indicates a more stable genetic correlation structure across ages in males, characterized by consistently positive correlations throughout the growth period. In contrast, genetic correlations between hatch weight (w_0_) and subsequent body weights in females were generally weaker and occasionally negative. Nevertheless, these sex-specific differences warrant cautious interpretation due to the high sampling variance of the estimates in both groups, and particularly within the male group (where standard errors of genetic correlations ranged from 0.28 to 0.68; results not shown).

### 3.5 Additive genetic covariance eigenfunctions

**Figure 4:**
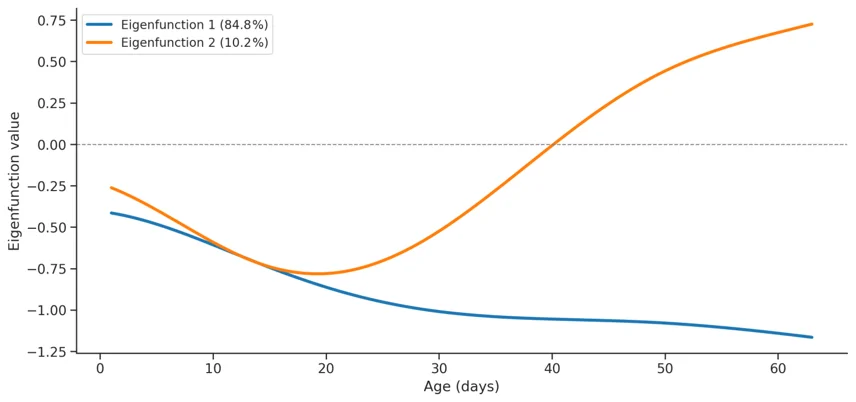
First two additive genetic variance eigenfunctions.

Figure 4 shows the first two additive genetic variance eigenfunctions, explaining jointly 95 % of the total genetic variance. The first eigenfunction (blue line) accounted for most of the additive genetic variation and remained relatively constant across ages, indicating the presence of a general genetic growth factor affecting body weight uniformly throughout development. This component can be interpreted as a scale effect, where some individuals are genetically predisposed to maintain consistently higher body weights across all growth stages. In contrast, the second eigenfunction (orange line) changed sign across age, shifting from negative values at early growth stages to positive values at later ages. This pattern suggests a genetic trade-off between early and late growth performance, reflecting differences in growth timing or developmental allocation strategies among individuals.

### 3.6 Effect of hypothetical selection at different growth stages

**Figure 5:**
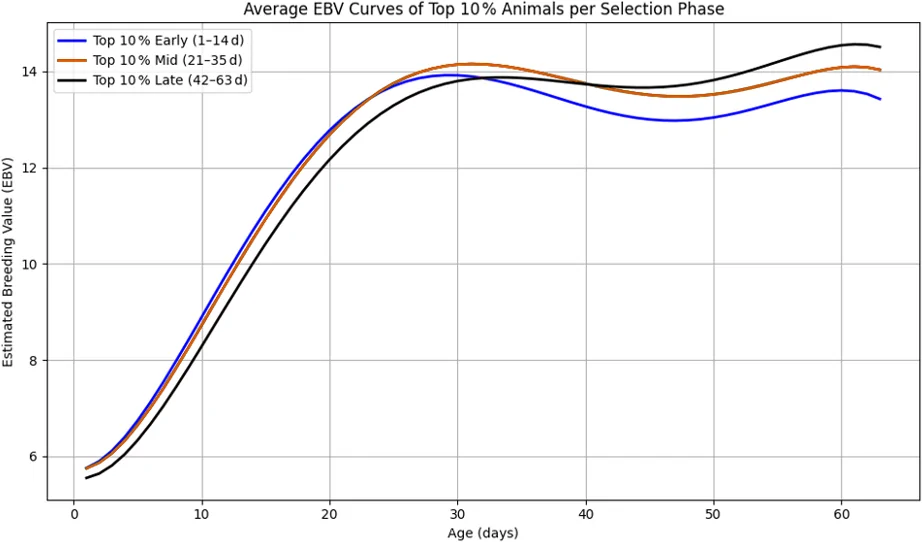
EBV curves of 10 % top animals selected at early (1–14 d; blue line), middle (21–35 d; orange line), and late age (42–63 d; black line).

The consequences of applying selection at different stages of growth are illustrated in Fig. 5. Note that the top 10 % selection pool ( n=68 ) per growth phase was derived from the 681 pedigree animals (out of 705 total) with available EBVs, including both phenotyped birds ( n=575 ) and unrecorded relatives. Selection based on EBVs during the early growth period resulted in high genetic merit at young ages, followed by a plateau or slight decline after the intermediate stage. This pattern suggests that birds selected for superior early growth tend to exhibit rapid juvenile development but do not necessarily maintain a proportional growth advantage later in life. In contrast, selection during the intermediate growth period produced more balanced EBV trajectories, with favourable genetic performance expressed throughout both the early and late stages of development. Selection based on EBVs at later ages generated a different pattern, characterized by relatively modest EBVs during the juvenile period followed by a continuous increase toward the end of the growth trajectory. These birds exhibited slower early growth but possessed greater genetic potential for achieving high final body weight.

**Figure 6:**
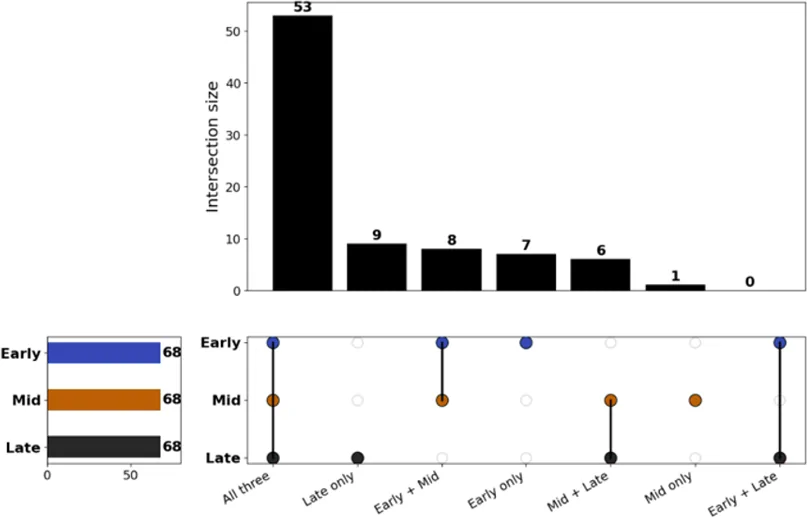
UpSet plot showing the overlap among animals ranked within the top 10 % based on phase-specific estimated breeding values (EBVs) during the early (1–14 d), intermediate (21–35 d), and late (42–63 d) growth phases. Upper bars represent the number of animals within each intersection, while the matrix below identifies the corresponding phase combinations. Coloured horizontal bars on the left indicate the size of each selected group.

Figure 6 presents an UpSet plot illustrating the overlap among animals ranked within the top 10 % based on phase-specific estimated breeding values (EBVs) during the early, intermediate, and late growth phases. Each phase-specific selection group contained 68 animals (top 10 % of the 681 animals with EBV estimates). In total, 84 unique animals appeared in at least one of the three top 10 % selection groups, indicating substantial overlap among phase-specific selections. The largest intersection comprised 53 animals that were simultaneously ranked among the top animals in all three growth phases, corresponding to 77.9 % of each phase-specific selection group. This substantial overlap indicates a high degree of stability in EBV rankings throughout development and suggests the presence of a large group of birds with consistently superior genetic merit.

Nevertheless, several animals exhibited phase-specific superiority. Eight animals were selected in both the early and intermediate phases but not in the late phase, whereas six animals were common to the intermediate and late phases only. Seven, one, and nine animals were uniquely selected in the early, intermediate, and late phases, respectively, while no animals were shared exclusively between the early and late phases.

Overall, these results demonstrate that although a large proportion of top-ranked animals maintain superior genetic merit throughout development, a smaller subset exhibits age-specific genetic advantages that may be exploited when selection objectives target particular growth stages.

## 4 Discussion

### 4.1 Model order and fit

The current study confirms the value of LP-based RRMs in modelling longitudinal growth with heterogeneous residual variance structures in the Japanese quail. A fifth-order polynomial was identified as optimal for weekly BW from hatch to 9 weeks, providing the best balance between goodness of fit and model complexity. The selected model adequately captured the major changes in growth dynamics, particularly the transition from rapid juvenile growth to the later stabilization phase, whereas lower-order polynomials tended to oversmooth the trajectory. Although the sixth-order model produced slightly better fit statistics, the improvement was marginal relative to the increased parameterization, suggesting limited biological and practical benefit. Similar findings have been reported in previous studies. Pavan et al. (2024) recommended fifth- and sixth-order polynomials for additive genetic and permanent environmental effects, respectively, in meat-type quail. Akbaş et al. (2004) and Gonçalves et al. (2012) favoured sixth-order models, with Gonçalves also incorporating six residual variance classes, and Bonafé et al. (2011) found that higher-order models generally improved model fit. In contrast, Karami et al. (2017) and Alkan et al. (2012) adopted more parsimonious third-order models, and studies by da Costa Caetano et al. (2017) and Mota et al. (2018) demonstrated that second- to fourth-order polynomials could also provide adequate fit, particularly when combined with heterogeneous residual variances. These findings suggest that the optimal polynomial order depends on the structure of the dataset and the complexity of the growth trajectory being modelled.

### 4.2 Heritability and $c^{2}$ across ages

The RRM revealed an age-specific trend in $h^{2}$ estimates, ranging from 0.25 at hatch, peaking at day 21 ( $h^{2}\approx 0.30$ ), and declining thereafter to approximately 0.14–0.15 at later ages. The peak in heritability around day 21 may reflect a stage at which maternal and incubation-related influences have largely diminished, while additive genetic differences in growth potential become more fully expressed. The subsequent decline may result from the increasing contribution of cumulative environmental effects and individual variation in resource allocation during later growth stages. Similar time-dependent patterns have been reported in Japanese quail, although the shape of the trajectory varies among studies. Akbaş et al. (2004) observed an increase in $h^{2}$ up to week 4, followed by a moderate decline, whereas Gonçalves et al. (2012) and El-Attrouny et al. (2020) reported a progressive decrease throughout growth. In contrast, Alkan et al. (2012) and Karami et al. (2017) found heritability to increase with age. Intermediate patterns characterized by a mid-growth peak followed by a decline, similar to the present results, were reported by Bonafé et al. (2011) and Pavan et al. (2024). In meat-type quail, Mota et al. (2018) and Silva et al. (2013) also reported moderate to high heritability estimates for body weight, with values generally declining after the period of rapid growth. Heritability estimates obtained from the MV analysis followed a similar overall pattern, although values tended to be slightly higher at later ages.

The relatively high estimates of permanent environmental effects estimated herein may reflect a broad set of non-additive and non-residual sources of variation that repeatedly affect the same individual across ages. In the present study, such effects may have originated from conditions established during the pre- and early post-hatch period, including egg-related maternal influences, incubation environment, early nutritional status, brooding conditions, social interactions, and cage-level management differences. These factors can create persistent differences among individuals that are maintained throughout the growth trajectory and may thus inflate the proportion of phenotypic variance attributed to permanent environmental effects. Consequently, the relatively high $c^{2}$ estimates highlight the importance of controlling early management conditions and, where possible, explicitly modelling maternal or common environmental effects in future studies.

### 4.3 Genetic and phenotypic correlations across ages

The present study revealed strong positive genetic correlations, particularly between adjacent time points, with a progressive decline in correlation strength as the temporal distance between ages increased. Similar patterns have been reported in previous studies on Japanese quail. Akbaş et al. (2004), Gonçalves et al. (2012), Karami et al. (2017), and Alkan et al. (2012) consistently observed high genetic correlations among adjacent ages and lower correlations as the interval between ages increased. An interesting finding of the present study was the negative genetic correlation between hatch weight and body weight at day 7 ( - 0.13), together with near-zero or low positive correlations between hatch weight and subsequent body weights. Similar results have been reported by Dionello et al. (2008) and Alkan et al. (2012), who also found negative genetic associations between hatch weight and later growth performance. These findings suggest that hatch weight may be under partly different genetic control than post-hatch growth and therefore may have limited value as a selection criterion for improving body weight at later ages. Moreover, hatch weight is known to be strongly influenced by maternal and early environmental effects (Faraji-Arough et al., 2022; Lotfi et al., 2012). Phenotypic correlations closely mirrored the genetic correlations, exhibiting higher values between adjacent ages and lower values between more distant ages. Similar trends have been reported by Akbaş et al. (2004), Gonçalves et al. (2012), Alkan et al. (2012), Silva et al. (2013), and Karami et al. (2017). In agreement with theoretical expectations, phenotypic correlations were generally lower than the corresponding genetic correlations (Roff, 1996).

Although both approaches produced broadly similar estimates, RRMs offer several practical advantages over MV analyses. RRMs require fewer parameters, provide smooth covariance functions across the trajectory, accommodate unequally spaced observations, and allow prediction of genetic parameters at intermediate ages. These properties make RRMs particularly attractive for routine genetic evaluation of longitudinal growth traits.

### 4.4 Sex-by-age specific genetic estimation

Heritability ( $h^{2}$ ) and permanent environmental effects ( $c^{2}$ ) showed some numerical differences between sexes across ages. Statistical analysis showed that $h^{2}$ estimates did not differ significantly, suggesting that the genetic contribution to body weight was generally similar in males and females throughout growth. Supporting this, studies in broilers using large datasets, such as the work of Maniatis et al. (2013) based on approximately 200 000 body weight records, also reported no sex-based differences in direct heritability estimates for body weight at 35 d of age. On the other hand, $c^{2}$ differed significantly between sexes, with higher values in males, especially during the early growth period. Such differences could reflect sex-specific sensitivity to environmental stress in early life, as males and females are known to respond differently in terms of behaviour, physiology, and gene expression. These variations may lead to longer-term effects on growth and other traits, especially in males (Elfwing et al., 2015). Genetic correlations were consistently higher in males but more variable in females. Nevertheless, due to the high sampling variance of estimates in the male group, the current findings should be interpreted with caution, and no general inference could be made.

### 4.5 Effects of growth stage selection and implications

To investigate the principal modes of additive genetic variation underlying growth in Japanese quails, eigenfunction decomposition of the additive genetic covariance matrix was performed. This approach enabled a comprehensive characterization of the genetic architecture governing growth throughout the developmental period. The identification of two biologically meaningful eigenfunctions indicated that body weight development is influenced by multiple genetic processes, including overall growth potential and age-specific patterns of growth expression. The first eigenfunction described uniform genetic variation across the entire growth trajectory, effectively distinguishing genetically fast- and slow-growing individuals at all ages. In contrast, the second eigenfunction revealed antagonistic genetic effects between early and late growth, indicating age-dependent differences in the expression of genetic potential. These results suggest that some individuals are genetically predisposed to rapid juvenile growth, whereas others exhibit greater genetic potential for growth during later developmental stages. Consequently, selection conducted at different ages may target partially distinct genetic mechanisms. Similar age-dependent growth patterns and early–late growth trade-offs have been reported in beef cattle (Nobre et al., 2003) and sheep (Fischer et al., 2004).

The practical implications of these findings were explored through a hypothetical selection scenario based on the top 10 % of birds ranked according to estimated breeding values (EBVs) within early, intermediate, and late growth stages. Selection during early growth may reduce generation interval and recording costs while achieving appreciable genetic gain. However, the corresponding EBV trajectories indicated that selection at young ages may preferentially identify birds exhibiting rapid juvenile growth without necessarily maintaining superior performance later in life.

Selection during the intermediate growth stage (21–35 d of age) produced more balanced EBV profiles across the entire growth trajectory, suggesting that this period represents a favourable compromise between early growth performance and sustained later development. In contrast, selection at later ages yielded the greatest expected response for final body weight but would require longer evaluation periods and increased maintenance costs before selection decisions could be implemented.

Therefore, the optimal age for selection depends on the breeding objective. Programmes aiming to improve early market weight and accelerate genetic turnover may benefit from earlier selection, whereas breeding schemes focused on maximizing mature body weight may require selection at later ages. Overall, the present results suggest that selection between 21 and 35 d of age offers the most advantageous balance among prediction accuracy, overall growth response, and generation interval, making this interval a particularly attractive selection window for practical breeding programmes. In conclusion, the application of the RRM facilitated the estimation of age-specific EBVs throughout the growth trajectory, providing greater flexibility in identifying superior animals and optimizing the timing of selection decisions.

### 4.6 Study limitations

This study was not without limitations. Potential confounding effects arising from non-modelled environmental sources of variation, such as micro-environmental heterogeneity within cages, as well as maternal genetic and permanent environmental effects, were not considered and may have influenced parameter estimates, particularly during early growth (Faraji-Arough et al., 2022; Lotfi et al., 2012; Maniatis et al., 2013). Future studies could benefit from explicitly accounting for these factors or incorporating genomic information to improve the accuracy of genetic parameter estimation. In addition, although LPs provided a flexible framework for modelling growth trajectories, alternative basis functions, such as B-splines, may also be worth investigating (Mota et al., 2018). Furthermore, the relatively moderate population size and the limited depth of the pedigree may have constrained the precision of variance component estimates and the ability to fully capture long-range genetic relationships. Deeper pedigrees, larger populations, or genomic relationship matrices could provide more accurate estimates of genetic parameters and breeding values.

### 4.7 Conclusion

In conclusion, a fifth-order LP RRM provided the best fit for quail growth trajectories. Eigenfunction analysis revealed a general growth factor and an antagonism between early and late growth. Selection between 21 and 35 d of age produced the most balanced genetic response across the growth trajectory, suggesting this period as an optimal selection window for Japanese quail breeding programmes.

## Data Availability

The data that support the findings of this study are available from the corresponding author upon reasonable request.
